# Two-substrate enzyme engineering using small libraries that combine the substrate preferences from two different variant lineages

**DOI:** 10.1038/s41598-024-51831-z

**Published:** 2024-01-14

**Authors:** Arka Mukhopadhyay, Kersti Karu, Paul A. Dalby

**Affiliations:** 1 Department of Biochemical Engineering, UCL, Bernard Katz Building, Gower Street, London, WC1E 6BT UK; 2 Department of Chemistry, UCL, 20 Gordon Street, London, WC1H 0AJ UK

**Keywords:** Protein design, Biotechnology

## Abstract

Improving the range of substrates accepted by enzymes with high catalytic activity remains an important goal for the industrialisation of biocatalysis. Many enzymes catalyse two-substrate reactions which increases the complexity in engineering them for the synthesis of alternative products. Often mutations are found independently that can improve the acceptance of alternatives to each of the two substrates. Ideally, we would be able to combine mutations identified for each of the two alternative substrates, and so reprogramme new enzyme variants that synthesise specific products from their respective two-substrate combinations. However, as we have previously observed for *E. coli* transketolase, the mutations that improved activity towards aromatic acceptor aldehydes, did not successfully recombine with mutations that switched the donor substrate to pyruvate. This likely results from several active site residues having multiple roles that can affect both of the substrates, as well as structural interactions between the mutations themselves. Here, we have designed small libraries, including both natural and non-natural amino acids, based on the previous mutational sites that impact on acceptance of the two substrates, to achieve up to 630× increases in *k*_cat_ for the reaction with 3-formylbenzoic acid (3-FBA) and pyruvate. Computational docking was able to determine how the mutations shaped the active site to improve the proximity of the 3-FBA substrate relative to the enamine-TPP intermediate, formed after the initial reaction with pyruvate. This work opens the way for small libraries to rapidly reprogramme enzyme active sites in a plug and play approach to catalyse new combinations of two-substrate reactions.

## Introduction

Biocatalysts have long promised to play a major role in synthetic organic chemistry, offering potentially greener routes to high-value chemicals^1,2^. However, their highly-valued specificity and selectivity also leads to limitations in their exploitation as they are more difficult to adapt to new substrates than traditional catalysts^3^. The challenge is particularly acute for enzymes that use two substrates as their interactions with the enzyme can often occur at overlapping sites. As a result, few studies have reported the engineering of enzymes to modify both substrate specificities to access new products^4,5^.

The formation of asymmetric carbon–carbon bonds is a critical route in organic synthesis to access a variety of innovative and natural compounds that can be used as building blocks in additional synthesis^6–8^. Due to their great selectivity and specificity, asymmetric carbon–carbon bond-forming enzymes like transketolase (TK) (EC 2.2.1.1) have significant synthetic potential^9,10^. TK catalyses the reversible transfer of a C_2_-ketol unit from donor substrate d-xylulose-5-phosphate to aldose acceptor substrates of either d-ribose-5-phosphate or d-erythrose-4-phosphate in the pentose phosphate pathway of all cells^11,12^. This metabolic pathway is critical in the cellular production of nucleotides, amino acids, and fatty acids.

In recent years, TK variants have been demonstrated in the synthesis of complex carbohydrates and other high-value compounds, including l-erythrulose^13^, deoxysugars^14^, *N*-Aryl Hydroxamic Acids^15^, 7-keto-octuronic acid^16^. At large scale, TK has been used for the production of unusual sugars^17^. Wild-type TK enzymes tend to accept a relatively limited range of hydroxyaldehyde aldol-acceptors with strict (2*R*)-specificity^17,18^. Donor substrates are similarly limited as they require an oxo group adjacent to the scissile C–C bond, and also typically a C-1 hydroxyl group. Furthermore, they tend to prefer donor substrates with a D-threo configuration of C-3 and C-4 hydroxyls^19^. Wild-type TK enzymes also have a preference for substrates that are phosphorylated due to complementary positively charged side-chains at the active site entrance^19–21^. However, this is not an absolute requirement, and TK can accept β-hydroxypyruvate (HPA) as the ketol donor which is advantageous in biocatalysis as it renders the donor half-reaction quasi-irreversible through liberation of CO_2_. This has been particularly useful for *E. coli* TK which gives up to 30-fold higher specific activity using HPA compared to yeast and spinach orthologs^22^.

To broaden the synthetic capability of transketolase, and to make it more readily adaptable to new reactions, a toolbox of variants is desired that can accept a wider set of acceptor and donor substrates. TKs from various organisms have been subjected to extensive modifications through both rational engineering and directed evolution. *E. coli* TK has been engineered for improved and reversed enantioselectivity^23^, stability^24,25^, and to progressively broaden the aldol-acceptor substrate range to polar aliphatics^21^, non-polar aliphatics^23^. TK from *Geobacillus stearothermophilus* (TK_gst_) has also been similarly engineered using related variants^26–29^.

**Figure 1:**
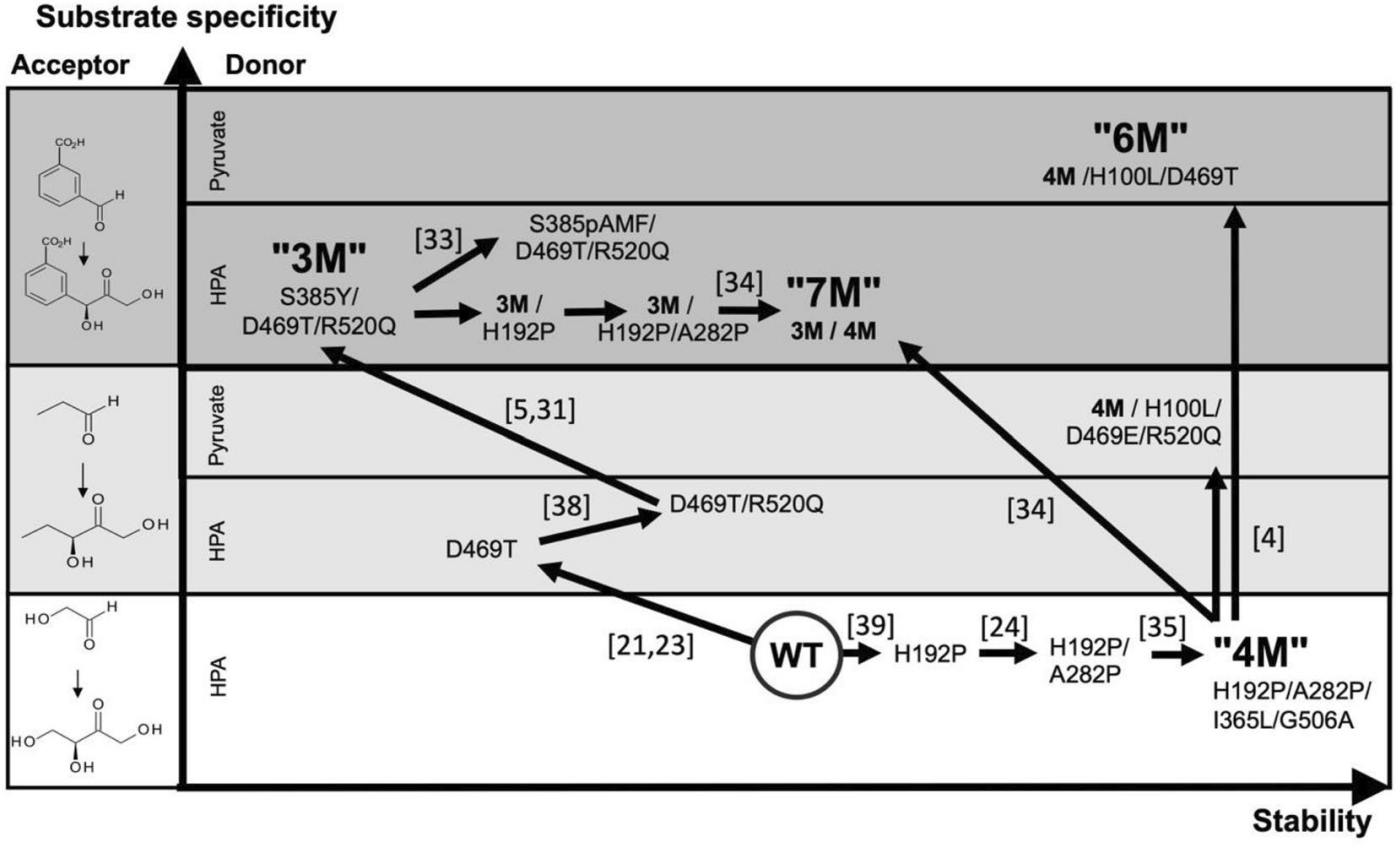
Lineages of *E. coli* TK variants. Multiple phases of rational, and semi-rational design guided by structure, bioinformatics and molecular dynamics simulations have introduced mutations that altered enzyme stability and the specificity for both aldol acceptor and ketol donor substrates. The 3M lineage first achieved activity on 3-FBA but with hydroxypyruvate as donor. The 6M lineage shown at the top right corner, was developed on acceptance of pyruvate as the donor, but achieved only low activity with 3-FBA. The current work explored combining the 3M and 6M lineages to improve activity of the reaction with 3-FBA and pyruvate.

One key goal has been to engineer *E. coli* TK to accept aromatic aldehydes, as shown through the lineage of engineered variants in Fig. 1. Initial work used structure-guided engineering of active-site residues to obtain the “3M” variant (S385Y/D469T/R520Q) that accepts 3-formylbenzoic acid (3-FBA) when using HPA as the ketol donor^5^. Crystal structural analysis of 3M and molecular docking of substrates revealed divergent binding modes^30^ for the three benzaldehyde analogues, 3-FBA, 4-formylbenzoic acid (4-FBA), and 3-hydroxybenzaldehyde (3-HBA). It was found that 3-FBA and 4-FBA oriented into two distinct binding pockets, whereas 3-HBA could bind into either with no obvious preference^30,31^.

The 3M variant has been further engineered to reverse the enatioselectivity^32^, while incorporating non-natural amino acids into the active site led to variants that were more stable, and more active towards 4-FBA and 3-HBA^33^. In the latter study, the variant **S385pCNF**/D469T/R520Q which incorporated *p-*cyanophenyl alanine at residue 385, was found to have 43× higher activity towards 3-HBA compared to the original 3M variant^33^. The stability of the 3M variant was also restored to wild-type levels using four previously known stabilising mutations to target residues within a network that was dynamically-correlated with active site residues^34^. The new “7M” variant, **H192P/A282P/I365L/**S385Y/D469T**/G506A**/R520Q retained its activity with 3-FBA but also improved the activity with 4FBA by 3×.

**Scheme 1:**
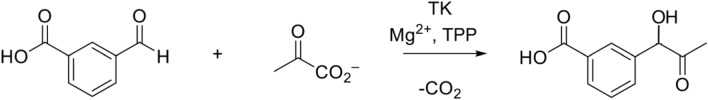
TK-catalysed synthesis of Phenylacetylcarbinol (PAC) analogue 3-(1-hydroxy-2-oxopropyl) benzoic acid from 3-formyl benzoic acid and pyruvate.

Another recent focus has been to engineer variants that can simultaneously use aromatic aldehydes with pyruvate as the ketol donor instead of HPA. This would give synthetic access to precursors to drugs such as spisulosine and phenylpropanolamine, and to analogues of phenylacetylcarbinol (PAC), an important pharmaceutical intermediate (Scheme 1). Pyruvate is only different from HPA by the absence of the C-3-hydroxyl group. However, it is unable to serve as a donor substrate in the TK process, highlighting the crucial significance of the hydroxyl group. The *E. coli* TK variant “6M” (**H100L**/H192P/A282P/I365L/D469T/G506A) was developed by introducing H100L and the previously known propanal-accepting D469T mutation into the WT-stabilising “4M” variant (H192P/A282P/I365L/G506A)^35^ to enable pyruvate acceptance as the donor along with propanal as the acceptor. A similar result was achieved recently with glycolaldehyde as the acceptor, by using a related pyruvate-accepting enzyme 1-deoxy-d-xylulose-5-phosphate synthase (DXS) to guide the engineering of TK_gst_ to accept pyruvate and other aliphatic analogues^36,37^.

Out of a series of variants, the “6M” *E.coli* TK variant was found previously to have the highest activity towards 3-FBA when using pyruvate, yielding just enough material in 48 h to isolate and characterise the product by NMR and LC/MS^4^. Interestingly, combining 3-FBA accepting mutations S385Y and R520Q from “3M”, into pyruvate accepting variants such as 4M/D469T/H473N and 4M/D469T/H473N/R520Q, led to losses in activity for the 3-FBA and pyruvate reaction^4^. The S385 and R520 residues both also form part of the entrance to the active site, and so their mutagenesis can impact not only on 3-FBA binding and orientation, but also may control the passage of pyruvate on its way towards the TPP cofactor.

Thus, the sites that influenced donor and acceptor substrate acceptance appeared to overlap or interact in a way that made combining beneficial mutations from each unpredictable. Furthermore, despite achieving success in obtaining activity with aromatic substrates and pyruvate, the activities obtained from the variants to date remained very low compared to those achieved with hydroxypyruvate (HPA) (Table 1).

**Table 1:** Specific activities of previous variants towards aromatic aldehydes.

Variant	Donor	Acceptor	*k*_cat_ (/s)	Specific activity (µmol/mg min)	References
3M: S385Y/D469T/R520Q	HPA	3-FBA	7.0	2.3	^5^
3M: S385Y/D469T/R520Q	HPA	4-FBA	n.d.	1.5	^5^
3M: S385Y/D469T/R520Q	HPA	3-HBA	n.d.	0.09	^5^
S385pCNF/D469T/R520Q	HPA	3-HBA	0.6	0.047	^33^
6M: H100L/H192P/A282P/I365L/D469T/G506A	PA	3-FBA	0.2	0.048	^4^

To date mutations from “3M” had not been explored within the “6M” variant. Therefore, in this new study, we created a small library that recombined the 6M *E. coli* TK variant (H192P/A282P/I365L/G506A/D469T/H100L) with R520Q and a range of S385 mutations, including non-natural amino acids. These were then screened against reactions with pyruvate as donor and the three aromatic acceptor aldehydes 3-FBA, 4-FBA and 3-HBA. This led to new variants with significantly improved activities and higher levels of conversion. Kinetic analysis alongside molecular docking of the substrates into key variants revealed possible reasons for improved activities.

## Materials and methods

### Materials

All chemicals were obtained from Sigma-Aldrich, Merck, Germany. Non-natural amino acid, p-Amino l-phenylalanine (pAMF) and p-Cyano-l-Phenylalanine (pCNF) were obtained from Chem Cruz (Texas, USA).

Qiagen Ni-NTA column followed by Amicon ultra 10KD filter unit were used to purify and concentrate the proteins. Transketolase gene carrying plasmid pQR791 and suppression plasmid, pUltra were obtained from our lab master stock. For reverse phase HPLC (C18 HPLC) Agilent ACE5 C18 reverse-phase column (150 × 9 × 4.6 mm) was used in departmental Agilent 1200 HPLC, UPLC-SQD2 was used for Mass spectrometric analysis and NMR.

### Methodologies

#### Preparation of a TK mutant library

TK variants were prepared by site directed mutagenesis using the Quick-Change site directed mutagenesis kit (Agilent technologies). Variants were obtained by sequential mutation from the 6M TK variant (H100L/H192P/A282P/I365L/D469T/G506A). A detailed list of mutants and the primers used is shown in Table S1 (supplementary information). This library consists of 8 variants in total. For the variants containing one of the two non-natural amino acids (nnAA) pAMF and pCNF at residue 385, we mutated the site to amber stop codon TAG. The nnAA incorporation was carried out by a standard amber suppression technique where the pUltra plasmid, pULTRA-CNF was a gift from Peter Schultz (Addgene plasmid # 48215)^40^ carrying the pAMF/pCNF specific aminoacyl synthetase gene, was co-transformed with the pQR729 plasmid for TK gene expression, into an amberless *E. coli* strain^41^ TK was then overexpressed using media supplemented with pAMF or pCNF. The nnAA incorporation was confirmed by ESI–MS experiments.

#### Transketolase purification

The cell pellets from 100-mL cell cultures for each variant or wild type were resuspended using cofactor solution containing 2.4 mM ThDP, 9 mM MgCl_2_ and 50 mM Tris/HCl, pH 7.0. The suspended cells were then transferred to a 50 mL falcon tube for cell lysis by sonication, then centrifuged at 18,900 g for 15 min to collect the cell lysate supernatant. The protein expression level was assessed by 12% SDS/PAGE. TK with nnAAs were overexpressed using a 1 mM IPTG induction. The TK was C-terminally fused to a His6 tag, and so TK variants were purified using a Ni–NTA column. Purity was assessed by 12% SDS PAGE and the concentration measured using the Bradford method. Eluted holo-TK was buffer exchanged into 2.4 mM ThDP, 9 mM MgCl_2_ and 50 mM Tris/HCl, pH 7.0, and diluted to 0.15 mg/mL or 0.2 mg/mL prior to enzyme activity reactions.

#### TK activities with aromatic aldehydes

Reactions between aromatic aldehydes and sodium pyruvate were initiated by adding 1 volume of substrate solution containing 150 mM aldehyde, 150 mM Na pyruvate, 50 mM Tris/HCl, pH 7.0, 2.4 mM ThDP, 9 mM MgCl_2_, to 2 volumes of the 0.15 mg/mL enzyme solution, to give final concentrations of 50 mM aromatic aldehydes and 50 mM sodium pyruvate. After reaction at 25 °C for 24 h, 20 μL of reaction mixture was transferred to 380 μL of 0.1% TFA to quench the reaction. Samples were then analysed by RP-HPLC with an ACE5 C18 reverse-phase column (150 × 9 × 4.6 mm), UV detection at 210 and 250 nm, and a mobile phase at a flow rate of 1.0 mL/min, starting with 70% of A (0.1% TFA) and 30% of B (100% acetonitrile) for 7 min.

#### Enzyme kinetics

Holo-TK variants at 0.2 mg/mL in 2.4 mM ThDP, 9 mM MgCl_2_ and 50 mM Tris/HCl, pH 7.0 were combined 1:1 with 2× substrate solutions. Kinetic parameters were obtained at 50 mM pyruvate and a range of 25–150 mM 3-FBA, in final conditions of 0.1 mg/mL holo-TK, 50 mM Tris–HCl pH 7.0, 2.4 mM ThDP, 9 mM MgCl_2_, at 21 °C. Aliquots of 50 μL were quenched at various times within 24 h by adding 350 μL of 0.1% (v/v) TFA. Triplicate reactions were monitored using RP-HPLC as above. All data were fitted by non-linear regression in OriginPro9.0, using the Michaelis–Menten equation to determine the *K*_m_ and *k*_cat_ of wild-type TK and the variants.

#### NMR analysis

Product fractions were collected from multiple C18 RP-HPLC runs, pooled and concentrated using a vacuum centrifuge until > 1 mg was obtained. This was dissolved in D_2_O within NMR tubes. ^1^H-NMR spectra were recorded on Bruker Avance 400, 500, 600 and 700 MHz spectrometers at 25 °C, using the residual protic solvent stated as the internal standard. Chemical shifts are quoted in p.p.m. to the nearest 0.01 p.p.m.

#### Sample preparation for capillary LC–MS/MS

For mass spectrometry, the C18 HPLC mobile phase was changed to 0.1% formic acid and 100% acetonitrile, and product fractions collected, concentrated using vacuum centrifugation, and then solid phase extraction with a Waters C_18_ 100 mg cartridge (Waters, UK) used to remove trace salts. The C_18_ cartridge was preconditioned following application of 100 µL of each sample, then washed with 1 mL of water and the sample components then desorbed with 1 mL methanol by gravity. 20 µL of each sample was then injected into the LC–MS instrument.

#### Capillary LC–MS/MS analysis

The LC–MS/MS system consisted of a Vanquish Liquid Chromatography LC system (Thermo Fisher Scientific, UK) coupled to a heated electrospray (HESI) probe connected to a Q Exactive mass spectrometer (Thermo Fisher Scientific, UK). The chromatographic separation was achieved on a Hypersil GOLD reverse-phase column (100 mm long, 2.1 mm internal diameter, particle size 1.9 µm) at a flow rate of 200 µL/min using a mobile phase A was 0.1% formic acid in water and mobile phase B was 0.1% formic acid in 80% acetonitrile. An eight minute gradient was as follows: After 1 min with 5% B, the proportion of B was raised to 95% B over the next 5 min, and maintained at 95% B for a further 1 min, after within 6 s the proportion of B was changed to 5% for 1.9 min to equilibrate the Hypersil C18 column. The LC column eluent was directed to the HESI source of the Q Exactive mass spectrometer. The HESI probe was operated with a sheath gas flow at 25 psi, an auxiliary gas flow at 10 psi, a spray voltage at 3.50 V, a capillary temperature at 320 °C, a S-lens RF level at 55, an auxiliary gas heater temperature was set at 50 °C. The Q Exactive mass spectrometer was operated in a positive ion mode. MS data was acquired using a data-dependent acquisition mode, and operated at 70,000 mass resolution (full width at half-maximum height, FWHM definition), and the top eight most abundant singly charged ions in the 80–800 m/z range were selected for MS/MS. The automatic gain control for the Q Exactive was set to 300,000 ions, and the automatic gain control for the MS/MS in the ion trap was set at 25,000 ions. For MS/MS, the isolation width was set at 1.2 amu and the collision energy was 30%. MS/MS scans were acquired at a mass resolution of 17,500 at 200 m/z. Three MS/MS microscans for each precursor were accumulated. Dynamic exclusion was enabled, and selected ions were excluded for 20 s before they could be selected for another round of MS/MS.

#### Computational molecular docking

The structures of the variants H192P/A282P/I365L/G506A/H100L/D469T/S385Y (TK1_7M), H192P/A282P/I365L/G506A/H100L/D469T/S385Y/R520Q (TK2_8M), H192P/A282P/I365L/G506A/H100L/D469T/S385F (TK3_7M) and H192P/A282P/I365L/G506A/H100L/D469T/S3854pCNF/R520Q (TK5_8M) were obtained using SWISS-MODEL and 5HHT.pdb as the template, and the S3854pCNF mutation introduced using the SwissSidechain Pymol plugin. The enamine-ThDP intermediate present in previously docked crystal structures was aligned into the WT and triple mutant (5HHT). The 3D conformation of 3-FBA was obtained from the PubChem (NIH) database and converted from a .sdf into a .pdb file using Open Babel. Autodock Vina was used to dock 3-FBA into WT and mutant TKs with the grid centred at − 11.25 Å, 25.858 Å and 40.198 Å (in 5HHT.pdb) and the exhaustiveness of 24^42^. The grid sizes of 30 Å × 30 Å × 30 Å, was used for the docking of 3-FBA and includes the entire active-site while omitting other surface hydrophobic pockets. Three replicate dockings were carried out and the conformations with lowest energy were selected for analysis. To get a comparative view, TK-WT and the four variant structures were also docked with 3-FBA in Autodock 4.2^43^. The explorable space for docking was defined using the same grid as above. For each search, a Lamarckian genetic algorithm was run 200 times with a maximum number of 25 million energy evaluations. The ligand (3-FBA) was flexible whereas the enzyme remained rigid. Resulting poses were analysed and checked for molecular interaction (hydrogen bonding, hydrophobic interaction, π-π stacking) in PyMOL Molecular Graphics System (Schrödinger, USA) and Protein Plus (TuHH).

## Results and discussion

Our goal was to find *E. coli* TK variants with improved activity towards aromatic acceptor aldehydes 3-FBA, 4-FBA and 3-HBA and with pyruvate as the donor, starting from the best variant to date, 6M (H192P/A282P/I365L/G506A/D469T/H100L). The 3M variant (S385Y/D469T/R520Q) was previously found to accept aromatic aldehydes, but only with hydroxypyruvate as donor. Previous attempts to introduce S385Y into 4M/D469T/H473N or 4M/D469T/H473N/R520Q actually led to a loss in activity towards 3-FBA with pyruvate^4^. However, this mutation was not previously tested in 6M. Other mutations of S385, including non-natural substitutions within 3M (S385X/D469T/R520Q) were also found previously to improve the activity towards aromatic aldehydes, but again only when using HPA as donor substrate^33^. The R520Q mutation has had a stabilising effect in several variants, often also improving activity^5,31,38^, but it did not improve the activity towards 3-FBA when inserted into 4M/D469T/H473N previously^4^. Therefore, we were interested now to explore whether any combinations of the various S385X mutations and also R520Q, could improve the activity of 6M towards aromatic aldehydes and pyruvate.

### TK variant activities with aromatic substrates

A series of TK variants were generated, expressed, purified and then evaluated for their activities towards three aromatic aldehyde acceptor substrates at 50 mM, with 50 mM pyruvate as the donor substrate. The variant names, mutations and initial levels of conversion after 24 h, based on product peak areas as a fraction of total peak area for substrate and product, are shown in Table 2.

**Table 2:** Conversion @24 h for all variants for 3FBA, 4FBA and 3HBA.

Name	Mutations	Conversion @24 h (%)		
		3-FBA	4-FBA	3-HBA
WT		0	0	0
6M	4M+H100L+D469T	2.5	n.d.	n.d.
TK-1	6M+S385Y	61	15	41
TK-2	6M+R520Q/S385Y	61	15	27
TK-3	6M+S385F	62	9	42
TK-4A	6M+S385pAMF	0	0	0
TK-4C	6M+S385pCNF	58	18	40
TK5A	6M+R520Q/S385pAMF	59	46	47
TK5C	6M+R520Q/S385pCNF	62	19	0
TK-6	6M+R520Q/S385F	62	15	44

6M: H100L/H192P/A282P/I365L/D469T/G506A.

All enzymes purified. Reactions at 25 °C with 0.1 mg/mL TK, 50 mM Tris–HCl, 2.4 mM ThDP, 9 mM MgCl_2_, pH 7.0, and final concentrations of 50 mM aromatic aldehydes and 50 mM sodium pyruvate. 6 M reaction was at 0.07 mg/mL enzyme^4^.

Among all eight variants TK-1, TK-2, TK-3 and TK-5C showed the greatest conversion to product after 24 h, and compared to no conversion with TK-WT. TK-3 and TK-5C also showed the greatest conversion when using 4FBA. The level of conversion for 3HBA remained relatively low with all variants. In previous work, the 6M variant (4M/H100L/D469T) gave only a 2.5% conversion of 3-FBA after 24 h. This could be increased at 1.3 mg/mL enzyme to 46.8%, but was still lower than the 62.2% achieved with the new TK-3 variant. Given the greatest activities with 3-FBA we focused further characterisation on that substrate, selecting the four most prominent variants TK-1, TK-2, TK-3 and TK-5C. We also used the conversion of 3-FBA with each TK variant to isolate the product and confirm its identity as previously^4^ using a combination of LC–MS and NMR (Supplementary information, Figs. S1, S2, S3).

### Kinetic and stability studies of TK variants with substrate 3-FBA and pyruvate

The kinetic parameters for TK-1, TK-2, TK-3 and TK-5C were determined at 0.1 mg/mL enzyme and 50 mM pyruvate by varying the concentration of 3-FBA from 0 to 150 mM, and monitoring reactions in triplicate for up to 24 h. The kinetic parameters are shown in Table 3, alongside the specific activity at 50 mM 3-FBA, and the conversion at 24 h (taken from Table 2). For comparison, the parameters determined previously for 6 M are also shown, although the *K*_m_ for 3-FBA was not determined as that experiment varied pyruvate instead. It was not possible to obtain parameters for WT-TK as no activity was detected and so rates and rate constants are set to 0, while the *K*_m_ cannot be determined.

**Table 3:** Enzyme kinetics and stability of selected variants.

Variant	Mutations	*V*_m_ (mM/min)	*k*_cat_ (/s)	*K*_m_ (mM)	Specific activity at 50 mM 3-FBA (µmol/mg min)	Conversion @24 h (%)	*T*_m_ (°C)
WT		0	0	n.d.	0	0	57.8
6M^a^	4M+H100L+D469T	0.012	0.22 (0.01)	n.d.	0.048 (0.005)	2.5	n.d.
TK-1	6M+S385Y	11 (7)	134 (85)	220 (180)	20.3 (5)	61	59.2
TK-2	6M+R520Q/S385Y	9.7 (3)	118 (41)	530 (190)	8.3 (0.3)	61	61.4
TK-3	6M+S385F	3.5 (0.2)	43 (3)	82 (11)	13.3 (1.5)	62	65.1
TK-5C	6M+R520Q/S385pCNF	4.2 (1.1)	51 (13)	95 (45)	14.6 (3)	62	63.7

^a^Kinetic parameters for 6M (H100L/H192P/A282P/I365L/D469T/G506A) were published previously^4^, using 0.067 mg/mL enzyme and by varying pyruvate at 50 mM 3-FBA. *K*_m_ is for 3-FBA at 50 mM pyruvate. Errors (as SEM) are shown in parentheses.

Compared to 6M, all four variants demonstrated significant increases in specific activity, *V*_m_ and *k*_cat_. The specific activity of TK-1 in particular was 400× higher than for 6M, while the *V*_m_ was 900× greater. While the *k*_cat_ values for TK-1 and TK-3 were 2–3× higher than for TK-3 and TK-5C, their *K*_m_ values were correspondingly higher. The resulting *k*_cat_/*K*_m_ values were fairly similar for TK-1, TK3 and TK-5C at 0.52–0.6/s mM, with TK-2 at a lower value of 0.2/s mM. Clearly the two larger *K*_m_ values (for TK-1 and TK-2) exceeded the studied range of 3-FBA (up to 150 mM), resulting in the larger errors on their estimated values.

The stability of the variants was also measured using thermal scanning fluorimetry with intrinsic fluorescence as the probe. TK is already known to aggregate rapidly upon unfolding^44^, but comparisons can still be made between variants with consistent ramping protocols, and so an apparent thermal transition mid-point (*T*_m_) is estimated. The *T*_m_ for wild-type TK was 57.8 °C, and consistent with a previous measurement of 58.3 °C under similar conditions (0.1 mg/mL, 25 mM Tris–HCl, pH 7.5, 0.5 mM MgCl_2_, 0.05 mM TPP)^44^.

The variants all had increased *T*_m_ values, reaching 65.1 °C, and 63.7 °C for TK-3 and TK-5C respectively. TK-1 and TK-2 gave more modest increases to 59.2 °C and 61.4 °C respectively. It was previously shown that four of the mutations used within 6M increased the *T*_m_ over wild-type TK by 3.6 °C^35^, whereas their introduction into 3M increased the *T*_m_ by 3 °C^34^. These earlier experiments suggest that a significant part of the 1.4–7.3 °C increased stability in the new variants was likely to have already been present in 6M.

Overall, TK-3 was the most stable, had the lowest *K*_m_ for 3-FBA and performed well in terms of catalytic efficiency (*k*_cat_/*K*_m_) and conversion (62%). However, for biocatalysis under conditions where substrate could be added in excess over *K*_m_, then the *k*_cat_ and stability are more important factors. With that perspective, the variants TK-1 and TK-2 might be preferred for their elevated activity despite the slightly lower *T*_m_ values than TK-3.

It is worth comparing the impact of R520Q and S385X mutations here with their addition to previous variants. R520Q in TK-2 increased the *T*_m_ over TK-1 by 2.2 °C with no adverse impact on activity, consistent with its stabilising effects in previous studies. S385Y and S385F each had a significant impact on activity when added to 6M in the current study, leading to respective 630× and 200× increases in *k*_cat_ for the reaction with 3-FBA and pyruvate. This contrasts with the previous impact of adding S385Y into the D469T/R520Q variant (to form “3M”), which improved the catalytic efficiency towards 3-FBA and HPA by significantly decreasing the *K*_m_ for 3-FBA. Furthermore, when the S385Y mutation was incorporated into 4M/D469T/H473N and 4M/D469T/H473N/R520Q, it led to a loss of activity towards 3-FBA with pyruvate^4^. The contextual mutations were clearly very important for the effects of S385Y. S385F gave a lower *k*_cat_, but also lower *K*_m_ for 3-FBA, when compared to S385Y, which mirrored the same result in S385X/D469T/R520Q variants when tested on 3-HBA and hydroxpyruvate^33^.

### Computational docking of 3-FBA into TK-WT, 7M and 8M

To provide some structural insights into the effects of mutations on *k*_cat_ and *K*_m_, we used computational docking of the 3-FBA substrate into the active site of the TK WT, TK-1 (7M), TK-2 (8M), TK-3 (7M) and TK-5C (8M) enzymes containing the enamine intermediate expected after prior reaction of pyruvate with the ThDP cofactor. Docking of the 3-FBA was performed in Autodock 4.2 which allowed both pose clustering (based on energy and RMSD for each pose) and an analysis of the relative populations of poses obtained for each cluster, from a total of 30. To analyse each pose and cluster in more detail, we calculated the distance, d, between the TPP enamine carbanion and the C-atom of the –CHO group of 3-FBA, to approximate the likelihood that a given pose was catalytically productive (not accounting for dynamics or angle of attack).

**Figure 2:**
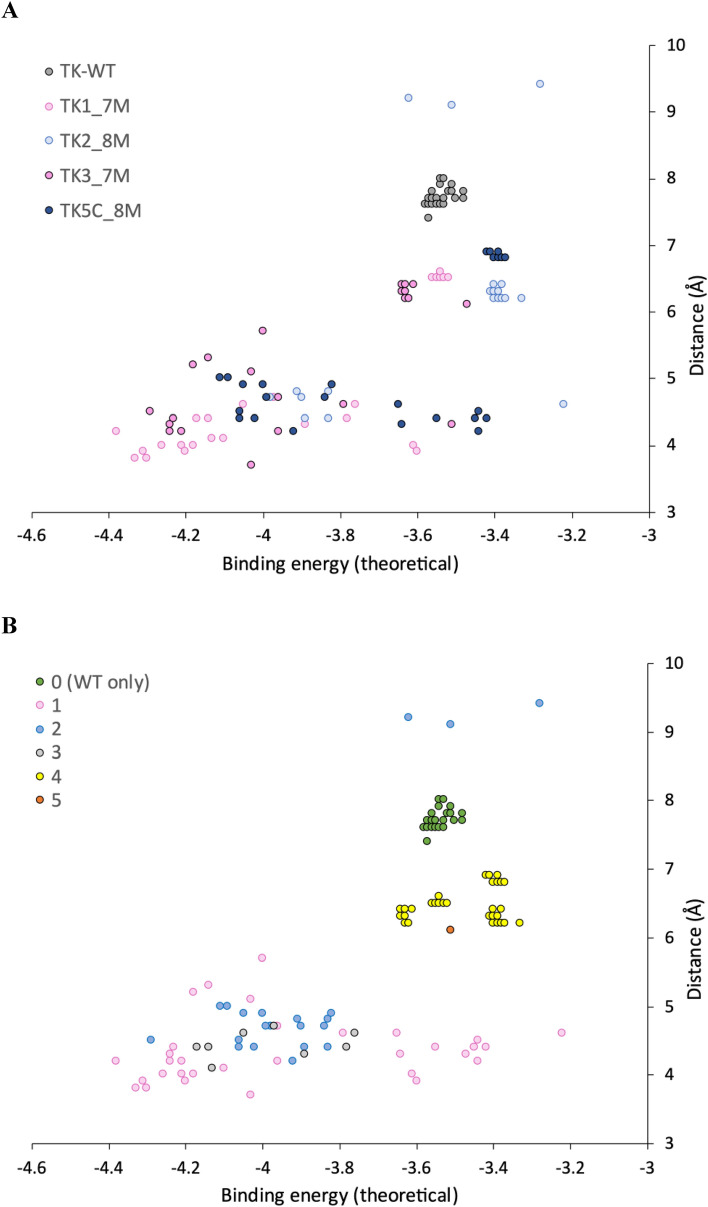
Distribution of binding energy and reactive distance for 3-FBA docking poses. Distributions are shown by (**A**) TK variant and (**B**) Docking cluster.

The poses for each variant are mapped in Fig. 2, and docking parameters shown in Table 4. The calculated cluster-averaged binding energies were all in the range − 3.2 to − 4.4 kcal/mol, indicating broad energetic similarity between substrates in all clusters such that all could potentially provide sufficient binding for catalysis. However, not all binding orientations and positions would necessarily be productive for catalysis, and significant populations of non-reactive complexes in the actual enzyme could contribute to either an increase in the apparent *K*_m_ or even lead to substrate inhibition. The maximum RMSD within each cluster varied from 0.1 to 1.0 Å, indicating a small combined degree of variance in substrate position, orientation and conformation within each cluster.

**Table 4:** Docking data for all variants with 3-FBA.

Variant	Mutations	Cluster	population (%)	Δ*G*_av_ (kcal/mol)	RMSD_max_ (Å)	d (Å)	Score
WT		0	100	− 3.54	0.45	7.7	7.0
TK-1	6M+S385Y	1a	3.3	− 4.38	0	4.2	76
		1	26.7	− 4.24	0.26	3.8	
		3	26.7	− 3.99	0.17	4.4	
		1b	6.7	− 3.61	0.19	4.0	
		4	36.7	− 3.54	0.19	6.5	
TK-2	6M+R520Q/S385Y	2	20.0	− 3.89	0.35	4.7	44
		2b	10.0	− 3.47	0.68	9.2	
		4	66.7	− 3.39	0.5	6.3	
		1b	3.3	− 3.22	0	4.6	
TK-3	6M+S385F	2	3.3	− 4.29	0	4.5	68
		1	46.7	− 4.04	0.96	4.2	
		4	46.7	− 3.63	0.11	6.4	
		5*	3.3	− 3.51	0	6.1	
TK-5C	6M+R520Q/S385pCNF	2	36.7	− 4.00	0.60	5	60
		1	23.3	− 3.51	1.0	4.3	
		4	40.0	− 3.40	0.11	6.9	

Population, average binding energy, RMSD, distance to ThDP-enamine, and docking score are given for each pose cluster.

Score = ∑ Pop_i_*(2–0.25*d_i_), limited 0–100% such that a distance of 4 Å or less scores as 100% while distances exceeding 8 Å score zero. RMSD_max_ is the highest RMSD for any pose within the cluster. *Cluster 5 only had a population of 1 pose across all variants.

The docking produced two or three distinct clusters for each of the variants, but only a single cluster for WT (Table 4). We also analysed all poses together by visualising within aligned structures to determine structural similarities and differences between clusters from each variant. This revealed that across the four mutant variants there were actually only four clusters in total, with a few outlying poses, and that these were all distinct from the only cluster (cluster 0) in WT. Cluster 0, found only in WT, and for all WT poses, was unreactive with 7.5 < d < 8 Å. Results from Autodock Vina were consistent with Autodock 4.2 (Average d = 7.2 Å), although.

Vina also identified a rare pose with d = 4.8 Å suggesting catalysis might be theoretically possible in WT-TK but achieving the correct substrate binding may be frustrated due to binding predominantly into a non-productive location.

Of the four clusters in variants, cluster 4 was unreactive with 6.1 < d < 7.0 Å. TK2 contained the fewest potentially reactive poses (23%), with 67% in cluster 4. A further 10% for TK2 were in cluster 2b, a subcluster of cluster 2 but with a 180° relative rotation in the plane of the 3FBA ring leading to d > 9 Å. Clusters 1, 2 and 3 could be considered as “reactive”. Cluster 1 was preferred by TK1 and TK3, (even though TK3 found a single pose in cluster 2 with a lower binding energy). TK1 and TK3 each retained the wild-type arginine at residue 520. By contrast, Cluster 2 was preferred by TK2 and TK5, and these two variants contained the R520Q mutation.

**Figure 3:**
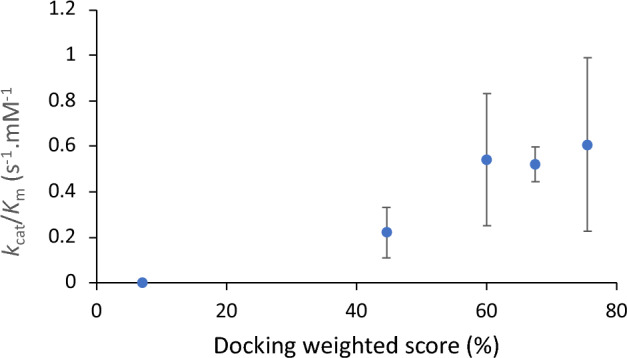
Correlation between *k*_cat_/*K*_m_ and docking weighted score. The docking score is a linear function derived from up to three clustered populations and their mean distance between enamine and acceptor carbonyl, as Score = ∑ Pop_i_*(2–0.25*d_i_), limited 0–100% such that a distance of 4 Å or less scores as 100% while distances exceeding 8 Å score zero.

To test the relationship between population, distance and experimentally determined catalytic efficiency, we created a score (Table 4) that weighted distance by population of poses, and linearly scaled between two cut-off values such that a distance of ≤ 4 Å scored 100% and ≥ 8 Å scored 0%. Given the limitations of docking methods this analysis does not account for dynamics or specific alignment of orbitals in favourable ways, such as at the Bergi-Dunitz angle for nucleophilic attack of a carbonyl^45^. However, intriguingly the score scaled well with *k*_cat_/*K*_m_ (Fig. 3), confirming that control of the distance to enamine, and population distributions were both major factors in the observed kinetic differences between the variants.

### How do mutations influence the binding populations?

**Figure 4:**
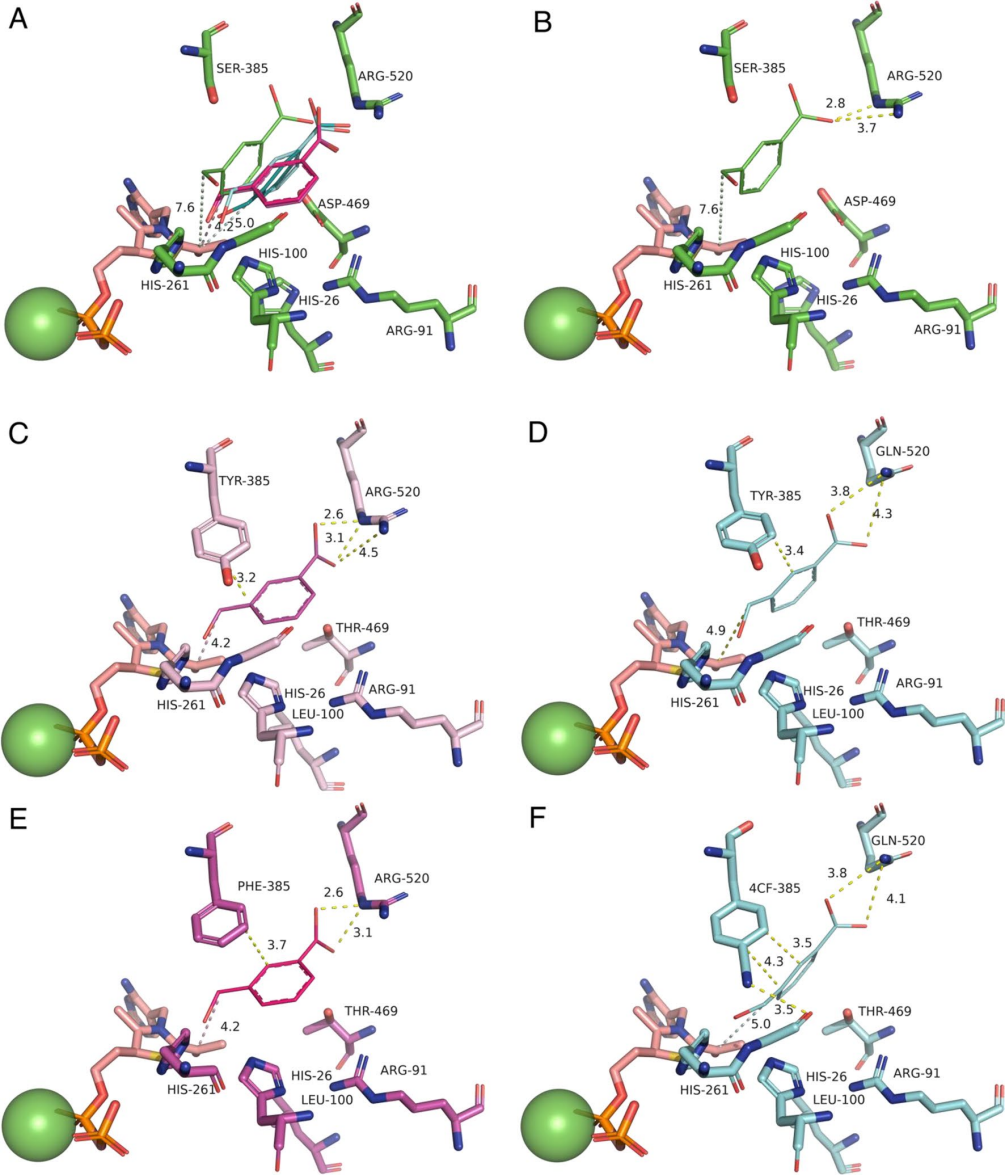
Alignment of the top docking poses for each TK variant and WT. Alignments show (**A**) all five top poses aligned within the WT structure, and then top poses in their respective structures for (**B**) WT; (**C**) TK-1; (**D**) TK-2; (**E**) TK-3; (**F**) TK-5. 3-FBA is represented as thick lines. TPP-enamine is shown as salmon sticks. Mg^2+^ cofactor is shown as green spheres. Docked 3-FBA is colour coded according the top structure in each cluster as (Green) Cluster 0 in WT only; (Magenta) Cluster 1; (Blue) Cluster 2.

Each pose was mapped to the active site structure by aligning them via their respective TPP-enamine cofactors, to then identify common interactions with the enzyme, and the potential role of mutations (Fig. 4). In previous studies, the WT enzyme was found to be inactive on the aromatic aldehyde substrate 3FBA^30,31^. The earlier 3M variant which included the S385Y and D469T mutations, first introduced activity towards 3-FBA in reactions with hydroxypyruvate^4,5^. A crystal structure of 3M and subsequent computational substrate docking of 3FBA, 4FBA and 3HBA, revealed that D469T mutation gave a hydrophobic surface for more favourable interactions with the nonpolar acceptor substrates^30^. The S385Y mutation meanwhile created opportunities for π–π stacking with the new substrates, but also sterically filled the active-site space to create a more defined binding pocket. Finally, the R520Q mutation decreased the potential for interaction with the carboxylate of 3-FBA or 4-FBA. Interestingly the docking in 3M previously showed two clusters arising from the formation of two distinct binding pockets with the aromatic ring positioned on either side of D469T. 3-FBA and 4-FBA oriented into different binding pockets, whereas 3-HBA could bind into either with no obvious preference. For 3-FBA the carboxylate group was oriented towards R91 to form a salt bridge interaction.

In the present study, docking of 3-FBA into the four key variants and WT, again revealed multiple potentially reactive binding modes, dominated by cluster 1 (Fig. 4C and E) and cluster 2 (Fig. 4D and F). Interestingly, the 3-FBA orientation observed in 3 M was no longer populated in the current variants, none of which made use of the previous interaction between 3-FBA and R91. Instead, the carboxylate groups remained oriented towards R520 in TK-1 (Fig. 4C) and TK-3 (Fig. 4E) as might be expected, but surprisingly also towards R520Q in TK-2 (Fig. 4D) and TK-5C (Fig. 4F). This orientation in TK-2 and TK-5C was supported through interactions with nearby H461 and to a lesser degree with R358, which were both also found in the WT docking of 3-FBA. However, the key difference to 3M appears to be the H100L mutation in the current variants. The imidazole sidechain of H100 interacts directly with the guanidinium of R91 in both WT-TK and 3M, and so the H100L mutation would directly modify the pKa for R91, most likely disfavouring ionisation and formation of a salt bridge to the 3-FBA substrate. This would explain the loss of the 3-FBA substrate orientation via R91 observed previously in 3M docking.

When comparing cluster 1 favoured by TK-1 and TK-3, to cluster 2 (preferred by TK2 and TK5), the key change was the R520Q mutation in TK2 and TK5. This mutation pulls 3-FBA slightly further away from the TPP-enamine (see distance d in Table 4), but also pulls it across the surface created by the D469T sidechain, thus enabling the aromatic ring face to rotate approximately 90 degrees and fit better into the cluster 2 binding pocket. By contrast, rotation of the 3-FBA ring face in TK-1 and TK-5, while maintaining the interaction with R520 would result in an unfavourable steric clash with the side chain of D469T.

## Conclusions

While earlier attempts to combine aromatic aldehyde-accepting mutations (S385Y, R520Q) into pyruvate accepting variants led to losses in activity, the current small library of S385X and R520Q mutations within the “6M” scaffold resulted in variants with up to 630× increases in *k*_cat_ for the reaction with 3-FBA and pyruvate. The best variants also retained enzyme stability as measured by their thermal transition midpoints. Variants with more modest 200× increases in *k*_cat_ had stabilities 4–5 °C higher. The *K*_m_ values of variants remained relatively high (80–530 mM) suggesting significant scope for further improvement, although in a biocatalytic process the aim would be to use high substrate concentrations that exceed the *K*_m_ values achieved.

This work demonstrates that rational recombination of mutations does not always combine their respective attributes, e.g. improved acceptance of new donor and acceptor substrates in the current case. However, by using small libraries based on the mutational sites identified previously, we can often find a successful solution to combining the multiple attributes.

Computational docking of substrates into the variant enzyme active sites explained the effects of the mutations in terms of shaping the active site pocket as well as in guiding the orientation of the aromatic aldehyde and its proximity to the enamine-TPP intermediate. The distance to the enamine achieved, weighted according to the population of poses found in each clustered position, gave a good correlation to the observed *k*_cat_/*K*_m_, indicating a strong role of that distance in improving enzyme activity, even before considering the more detailed effects of protein dynamics or specific orbital alignments.

### Supplementary Information

Supplementary Information.

## Data Availability

All data reported here are available on request to the corresponding author.
