# Supplementary material for: Two-substrate enzyme engineering using small libraries that combine the substrate preferences from two different variant lineages
Source: Sci Rep. 2024 Jan 14;14:1287. doi: 10.1038/s41598-024-51831-z (PMC10787763; doi:10.1038/s41598-024-51831-z)
Supplement: Supplementary file 1 — Supplementary Information. [file 41598_2024_51831_MOESM1_ESM.docx]

**Supplementary Information:**

**Two-substrate enzyme engineering using small libraries that combine the substrate preferences from two different variant lineages**

**Arka Mukhopadhyay, Paul A. Dalby**

Correspondence*: [p.dalby@ucl.ac.uk](mailto:p.dalby@ucl.ac.uk)

**Table S1:**

**Primers for site-directed mutagenesis:**

| **Mutations** | **Forward Primers** | **Reverse Primers** |
| --- | --- | --- |
| **S385amber** | 5’ GGCGCCGTAGAACCTGACCCTGTGGTCTGGTTC 3’ | 3’ AGGTTCTACGGCGCCAGGTCAGCAG 5’ |
| **S385Y** | 5’ GGCGCCGTATAACCTGACCCTGTGGTCTGGTTC 3’ | 3’ AGGTTATACGGCGCCAGGTCAGCAG 5’ |
| **S385F** | 5’ GCGCCGTT**C**AACCTGACCCTGTGGTCTGG 3’ | 3’ CAGGTTGAACGGCGCCAGGTCAGCAG 5’ |
| **R520Q** | 5’ CTCTCCCAGCAGAACCTGGCGCAGCAG 3’ | 3’ GTTCTGCTGGGAGAGGATCAGTGCGGTC 5’ |

**Scheme of TK reaction with 3FBA and NA-pyruvate:**

**
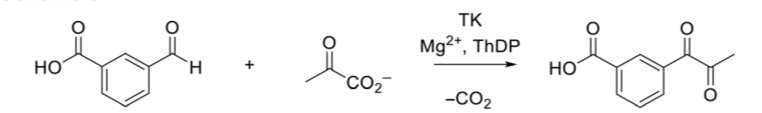
**

**3FBA Pyruvate 3-(1-hydroxy-2-oxopropyl) benzoic acid**

**C18 HPLC chromatography for substrate and product quantitation:**

All the 8 variants were screened for activity towards 3-FBA and sodium pyruvate as determined by C18 HPLC. After reaction for 24 h, a new peak at the retention time of 3.4 min was identified that correlated with the consumption of the 3-FBA in the HPLC chromatogram for 4 of the variants, but not for WT and TK4A, TK4C, TK5A and TK6

**
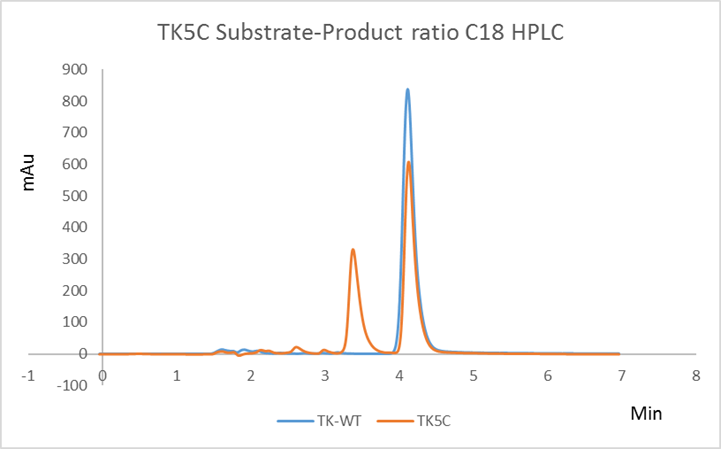

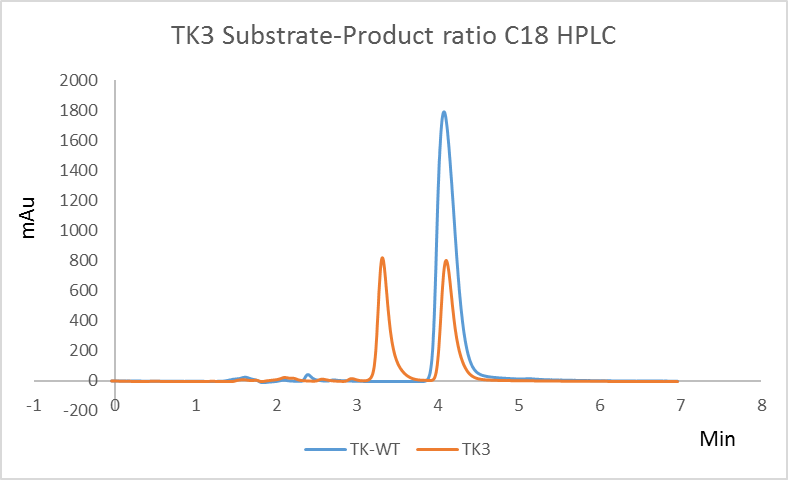

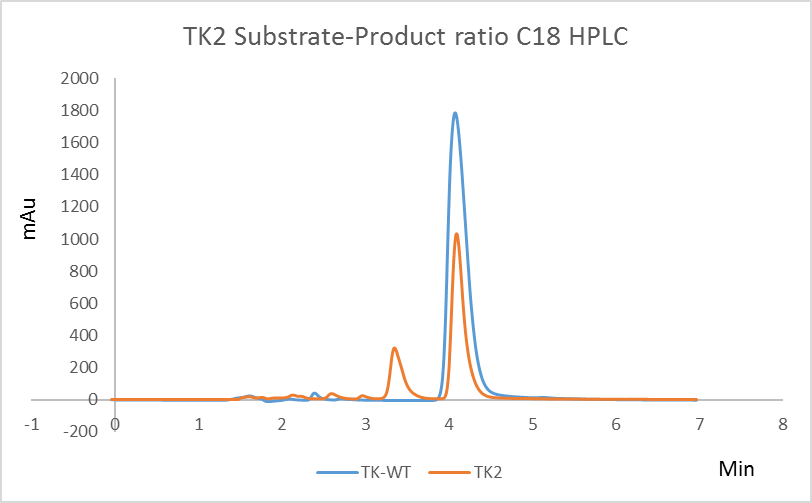
**

D

B

C

A

**
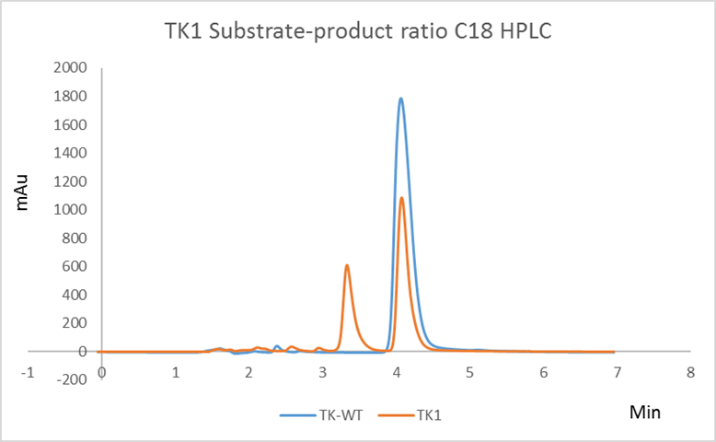
**

**Figure S1**: C18 HPLC chromatogram. Orange peak at 3.4 mins is the product peak and the orange and blue peaks at 4.1min is for the substrate 3FBA. A: TK1, B: TK2, C: TK3 and 4: TK5C.

**Structural confirmation of aromatic products:**

In order to confirm the identity of the product, the peak generated in C18 HPLC was isolated by semi preparative HPLC and characterized by 1H NMR spectroscopy and LC-MS.

**^1^H NMR spectroscopy:** In ^1^H spectroscopy, we found one singlet at 8.2ppm that clearly confirmed the presence of the benzene ring in our product. A triplet at 7.45ppm, doublets at 7.45ppm, 8.12ppm, three singlets at 2.0 ppm and one singlet at 3.5ppm confirmed the product C_10_H_10_O_4_ with the molecular structure shown and assigned in Figure S2


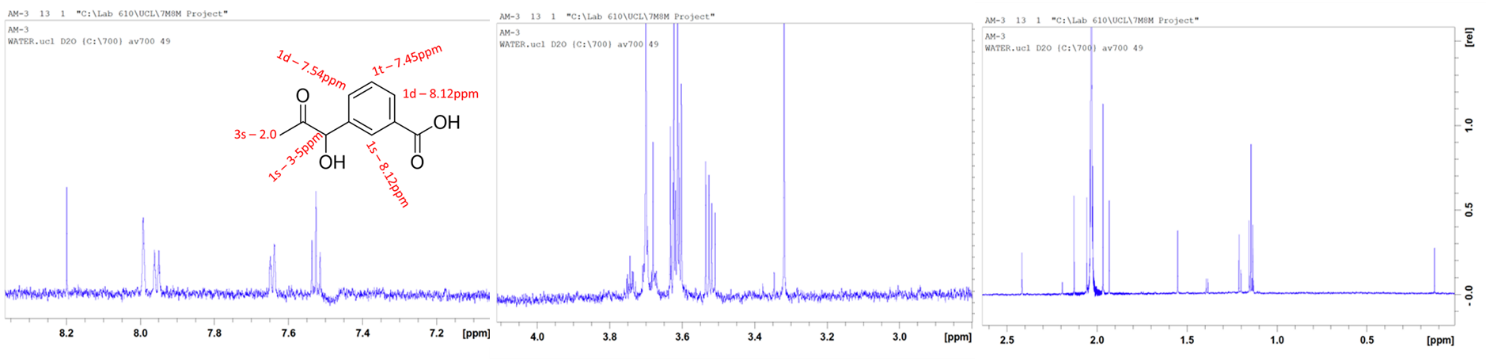


**Figure S2**: ^1^H NMR for 3-(1-hydroxy-2-oxopropyl) benzoic acid

**Capillary LC-MS analysis:**

The mass analyser was set to target *m/z* 195.0652 which corresponds to [M+H]^+^ species formed during HESI ionisation of the compound with an empirical formula C_10_H_10_O_4_. Therefore, we acquired mass spectrometric data in a full mass range *m/z* 50-600 and MS/MS at *m/z* 195.0652 during the LC-MS analysis. The reconstructed ion chromatogram (RIC) for *m/z* 195.06 shows two chromatographic peaks (Supplementary Figures). The chromatographic peak at 4.29 min displays a HESI mass spectrum with several peaks at *m/z* 195, an accurate measurement of the ion at *m/z* 195.0652 generated the elemental composition C_10_H_11_O_4_, which corresponds to [M+H]^+^ ion with the mass measurement error of 0.04 ppm. The isotopic signature of [M+H]^+^ ion also showed high accuracy, with <10% errors in relative isotopic abundance for C_10_H_11_O_4_. Figure 1 shows the MS/MS spectrum of *m/z* 195.06 corresponding to the chromatographic peak at 4.29 min. The [M-18]^+^ ion at *m/z* 177 corresponds to the loss of water from [M+H]^+^ ion. The fragment ion at *m/z* 149 [M-46]^+^ generated due to the loss of CH_2_O_2_ from [M+H]^+^ ion_._ Whereas the ion at *m/z* 121 [M-74]^+^ reflecting the loss of C_3_H_7_O_2_. The MS/MS spectrum shows an abundant fragment ion at *m/z* 137 [M-58+H]^+^ corresponding to the loss of C_2_H_2_O_2_ from the [M+H]^+^ ion. Therefore, the fragmentation pattern consisted with the proposed structure C_10_H_10_O_4_. (Figure S3)

*
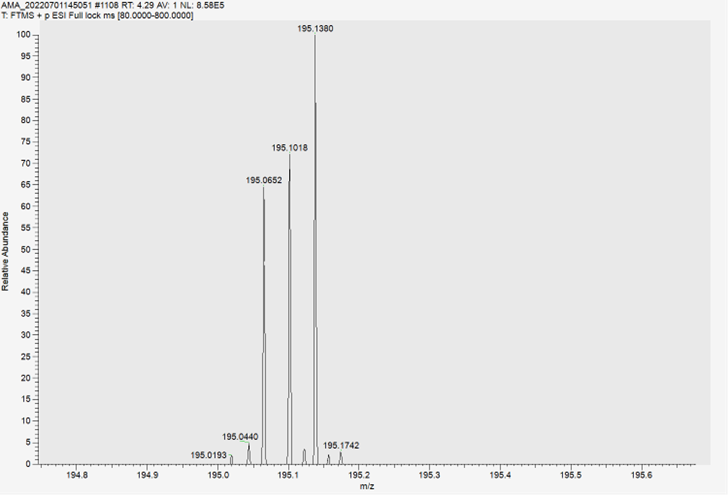
*
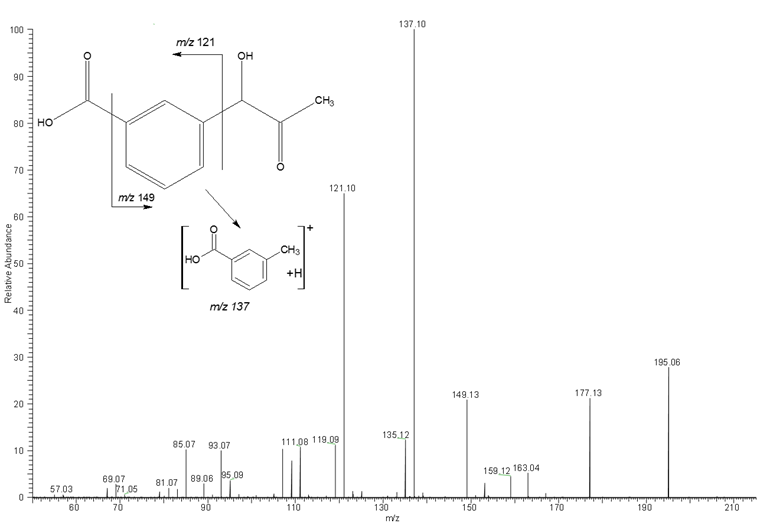


**Figure S3:** MS/MS for m/z 195 at retention time 4.32 min for Sample from TK1/2/3 and 5C
